# Enhanced recovery after surgery in elective cesarean section patients with gestational diabetes mellitus does not lead to glucose-related maternal and neonatal complications

**DOI:** 10.3389/fendo.2024.1403754

**Published:** 2024-08-06

**Authors:** Jin Zhou, Peizhen Zhang, Zhangmin Tan, Chuo Li, Lin Yao, Tiantian He, Hongyin Hou, Yuzhu Yin

**Affiliations:** Department of Obstetrics and Gynecology, The Third Affiliated Hospital of Sun Yat-sen University, Guangzhou, China

**Keywords:** enhanced recovery after surgery, gestational diabetes mellitus, cesarean section, hypoglycemia, carbohydrate

## Abstract

**Objective:**

For elective cesarean section patients with gestational diabetes mellitus (GDM), there is a lack of evidence-based research on the use of enhanced recovery after surgery (ERAS). This study aims to compare the ERAS after-surgery protocol and traditional perioperative management.

**Research design and methods:**

In this retrospective cohort study, singleton pregnancies with good glucose control GDM, delivered by elective cesarean sections under intravertebral anesthesia at least 37 weeks from January 1 to December 31, 2022, were collected at the Third Affiliated Hospital of Sun Yat-sen University. We divided all enrolled pregnant women and newborns into an ERAS group and a control group (the traditional perioperative management group) based on their adherence to the ERAS protocol. The primary outcome was the preoperative blood glucose level, with an increase of more than 1 mmol/L indicating clinical significance when compared to the control group. The secondary outcome was centered around an adverse composite outcome that affected both mothers and newborns.

**Results:**

We collected a total of 161 cases, with 82 in the ERAS group and 79 in the control group. Although the mean preoperative blood glucose level in the ERAS group was significantly higher than in the control group (5.01 ± 1.06 mmol/L vs. 4.45 ± 0.90 mmol/L, *p*<0.001), the primary outcome revealed that the mean glycemic difference between the groups was 0.47 mmol/L (95% CI 0.15-0.80 mmol/L), which was below the clinically significant difference of 1 mmol/L. For the secondary outcomes, the ERAS group had an 86% lower risk of a composite adverse outcome compared to the control group. This included a 73% lower risk of perioperative maternal hypoglycemia and a 92% lower rate of neonatal hypoglycemia, all adjusted by age, hypertensive disorder of pregnancy, BMI, gestational weeks, primigravidae, primary pregnancy, GDM, surgery duration, and fasting glucose.

**Conclusion:**

Implementing a low-dose carbohydrate ERAS in pregnant women with GDM prior to elective cesarean section, compared to traditional perioperative management, does not lead to clinically significant maternal glucose increases and thus glucose-related maternal or neonatal perioperative complications.

## 1 Introduction

Enhanced recovery after surgery (ERAS) application in cesarean deliveries has markedly enhanced maternal and neonatal outcomes and grown extensively in recent years (1–3). However, the feasibility of implementing ERAS in cesarean deliveries for patients with diabetes remains a subject of debate. A primary concern is that the carbohydrate intake prescribed in ERAS preoperatively could complicate glycemic control, leading to the exclusion of pregnant women with diabetes from the current ERAS-related guidelines (1–3).

Gestational diabetes mellitus (GDM) is prevalent, affecting approximately 14.8% (95% CI 12.8–16.7%) in China (4). Patients with GDM are at an increased risk of macrosomia and other complications, resulting in higher cesarean section rates, postoperative infections, and slower wound healing, which prolong hospital stays. Additionally, GDM, coupled with extended fasting before cesarean sections, can exacerbate perioperative complications, including maternal insulin resistance and maternal and neonatal hypoglycemia. Globally, there is a lack of evidence-based research on the use of ERAS in pregnant women with diabetes. Due to concerns that preoperative carbohydrate loading in ERAS may exacerbate hyperglycemia, many healthcare organizations currently do not recommend ERAS for diabetic patients (5). However, a prospective non-inferiority cohort study involving non-pregnant diabetic patients revealed that preoperative carbohydrate supplementation yielded outcomes comparable to fasting, with no significant differences in preoperative glucose levels, hyperglycemia, or hospital stay duration (6). For GDM patients undergoing caesarean sections, administering a low-concentration carbohydrate solution two hours prior to surgery has proven to be a safe and viable option. It is efficacious in reducing the incidence of preoperative hypoglycemia and improving patient health without increasing the risk of maternal hyperglycemia and neonatal hypoglycemia (7). Various clinical trials have assessed carbohydrate supplementation or feeding during labor for improved labor outcomes (8). In GDM patients, small-dose carbohydrate preconditioning appears safe.

This study hypothesizes that, compared to traditional perioperative management, ERAS (including small-dose carbohydrate) implementation in cesarean deliveries for women with GDM will not result in challenging glycemic control or significant glucose-related safety concerns for the mothers and their newborns. Previous studies have found that preoperative glucose levels, linked to postoperative complications and neonatal hypoglycemia in diabetic patients (9–11), may act as evaluation metrics for surgical complications (6). A 1 mmol/L increase beyond the normal range in maternal blood glucose raises the risk of neonatal hypoglycemia (9). Consequently, our primary study outcomes were preoperative glucose, which was a proxy for maternal glucose-related perioperative complications. A preoperative glycemic difference value >1 mmol/L compared to the control can be regarded as clinically significant. Meanwhile, composite adverse outcomes, including maternal and neonatal glycemic abnormalities and corresponding adverse effects, were defined as secondary study outcomes. We used multivariate regression analysis to assess whether ERAS increases the risk of adverse glucose-related perioperative complications in pregnant women with GDM compared to the control group (traditional perioperative management).

## 2 Materials and methods

The study received approval from the Institute Medical Ethics Committee of the Third Affiliated Hospital of Sun Yat-sen University (reference number: [2021] 02-277-01), and the need for informed consent was waived due to the retrospective nature of the study. The study adhered to the Strengthening the Reporting of Observational Studies in Epidemiology (STROBE) reporting guidelines for the retrospective cohort studied (12).

In this retrospective cohort study, data were collected on expectant individuals admitted to the obstetrics department through the hospital’s electronic medical record system at the Third Affiliated Hospital of Sun Yat-sen University from January 1 to December 31, 2022. The inclusion criteria were patients with singleton pregnancies diagnosed with GDM, managed effectively through diet (GDMA1) or hypoglycemic agents (GDMA2) (13) (HbA1c<5.5%), at least 37 weeks of gestational age, and scheduled for elective cesarean sections under intravertebral anesthesia. Conversely, the exclusion criteria encompassed emergency cesarean sections, use of general anesthesia, history of gastric emptying obstruction, severe cardiopulmonary abnormalities, autoimmune diseases, active infections, pre-pregnancy diabetes, psychiatric or psychological disorders impeding dietary compliance, and severe fetal anomalies. Intraoperative changes in anesthesia or postoperative transfer to the intensive care unit should also be excluded. Additionally, all selected pregnant women and newborns were divided into two groups (the ERAS group and the control group) depending on whether they had taken the ERAS protocol.

### 2.1 Sample size calculation

Previous studies indicate that preoperative hyperglycemia, as a surrogate predictor of postoperative maternal and neonatal complications in patients undergoing GDM, significantly correlates with increased postoperative complications and neonatal hypoglycemia. A 1 mmol/L increase in maternal blood glucose above the normal range raises the risk of neonatal hypoglycemia (9). An increase of 1 mmol/L in maternal blood glucose within the normal range did not raise the risk of neonatal hypoglycemia. Consequently, we propose that there should be significant clinical differences when the average difference in maternal blood glucose is greater than 1 mmol/L. We retrospectively analyzed 458 GDM patients with optimal glycemic control who underwent elective cesarean sections in our hospital. These patients’ mean preoperative glucose level was 4.9 ± 0.9 mmol/L. This study’s significance level was set at α = 0.025, and the statistical power at 1-β = 0.9. Using PASS 11 software, we calculated the required sample size for both the ERAS and control groups to be approximately 77 cases each. Anticipating a 10% non-cooperation rate in the ERAS procedure, the sample size was adjusted to about 85 cases per group. Consequently, each group in this study, the ERAS and non-ERAS among GDM patients, comprised 85 cases.

### 2.2 Definition and implementation of ERAS and control protocols

The ERAS group followed an accelerated rehabilitation surgical program based on evidence-based recommendations (**Supplementary Table 1**). This program emphasized reduced fasting duration, early mobilization, initiation of a diet soon after surgery, and prompt catheter removal (refer to **Supplementary Table 1** for core components of the ERAS protocol at our institution). In contrast, the control group adhered to a routine service program, which included preoperative fasting post-dinner, flexible post-surgery bedrest, fasting on the day of surgery, a fluid diet following the first postoperative venting, and catheter removal 24 hours post-surgery.

A key element of ERAS is minimizing preoperative fasting to mitigate the insulin resistance that often accompanies prolonged fasting, representing a significant change in practice at our institution. The preoperative dietary protocol for pregnant women enrolled in ERAS (including those with and without gestational diabetes mellitus, GDM) entailed: an 8-hour fast from meats, fats, and solid foods; a 6-hour fast from starches and dairy products; oral intake of 52 g Glucerna nutritional formula powder (comprising 21.15 g protein, 15.38 g fat, and 55.90 g carbohydrate per 100 g) by midnight before surgery; and consumption of 300 ml of a clear, carbohydrate-rich drink (14.2% carbohydrate, containing 42.6 g carbohydrate, produced by Yichang Renfu Pharmaceutical Co., Ltd.) two hours before surgery. Total carbohydrate intake below 50 g was deemed a low dose (14), as per a referenced meta-analysis. We utilized a clarified beverage containing 42.6 g of carbohydrate with maltodextrin, enhancing intestinal transport mechanisms and thereby expediting energy absorption and hydration while reducing gastric emptying time (15). This preparation also alleviates patient discomfort, including thirst, hunger, and anxiety (16). In contrast, the control group fasted after the previous evening’s meal and continued intravenous rehydration from two hours preoperatively until the start of the caesarean section (CS). Rehydration was performed after CS until anal exhaust or defecation, and the gestational women were instructed to get out of bed 1-2 days after the CS and have liquid food after anal exhaust.

### 2.3 Observational indicators and methods

The primary outcome measured was the preoperative blood glucose level (mmol/L) in GDM patients. The secondary outcome focused on an adverse composite outcome for mothers and newborns, covering maternal perioperative hypoglycemia and hyperglycemia, neonatal hypoglycemia, low Apgar scores, abnormal PH in umbilical artery blood gas, and immediate post-delivery transfer to the pediatric unit, with any abnormality marking a positive result.

Maternal blood glucose monitoring: This included fasting blood glucose (fingertip) on the day of surgery, preoperative blood glucose (fingertip), immediate postoperative blood glucose (fingertip) after surgery before patient departure from the operating room, and fasting blood glucose (fingertip) on the day following surgery.

Neonatal blood glucose testing: neonates in both groups underwent hourly fingertip blood glucose measurements for four hours post-birth, with the minimum recorded value representing neonatal blood glucose.

Maternal perioperative hypoglycemia was defined as perioperative blood glucose levels falling below 3.3 mmol/L at any point; perioperative hyperglycemia was identified when blood glucose levels were 7.8 mmol/L or higher (17). Severe hyperglycemia was characterized by blood glucose levels exceeding 10.0 mmol/L (17). Neonatal hypoglycemia was defined as a blood glucose level below 2.6 mmol/L within the first four hours post-birth, as per the Queensland Guidelines 2022 (18).A neonatal low Apgar score was indicated by a score of 7 or less within 5 minutes of birth, and neonatal blood gas abnormalities were marked by an umbilical artery blood gas PH below 7.2 (19).

### 2.4 Statistical analysis

Measurements were analyzed using the statistical software R 4.2.2 (http://www.R-project.org. R Foundation) and the Free Statistics software platform (Beijing, China), with results expressed as mean ± standard deviation for normally distributed data or interquartile range for non-normally distributed data. We conducted group comparisons using the t-test for normally distributed data, the Wilcoxon rank-sum test for non-normally distributed data, and the chi-square test for count data. The primary study outcome was assessed for differential maternal preoperative glucose using a one-sided 95% confidence interval by linear regression, with differences considered to be significant when the average difference was greater than 1 mmol/L with a P-value of less than 0.025. And this model adjusted for maternal age, hypertensive disorder of pregnancy (HPD), body mass index (BMI), gestational age, primigravidae, GDM, caesarean section (CS) time, and fasting glucose. For the secondary study outcomes, a multivariate regression model was employed to examine the impact of ERAS on the composite glycemia and delivery outcome in patients with GDM undergoing elective cesarean delivery. This model was adjusted for maternal age, HPD, BMI, gestational age, primigravidae, primiparous, GDM, CS time, and fasting glucose. A P-value of less than 0.05 was considered statistically significant.

## 3 Results

### 3.1 Baseline characteristics of the study population

**Figure 1:**
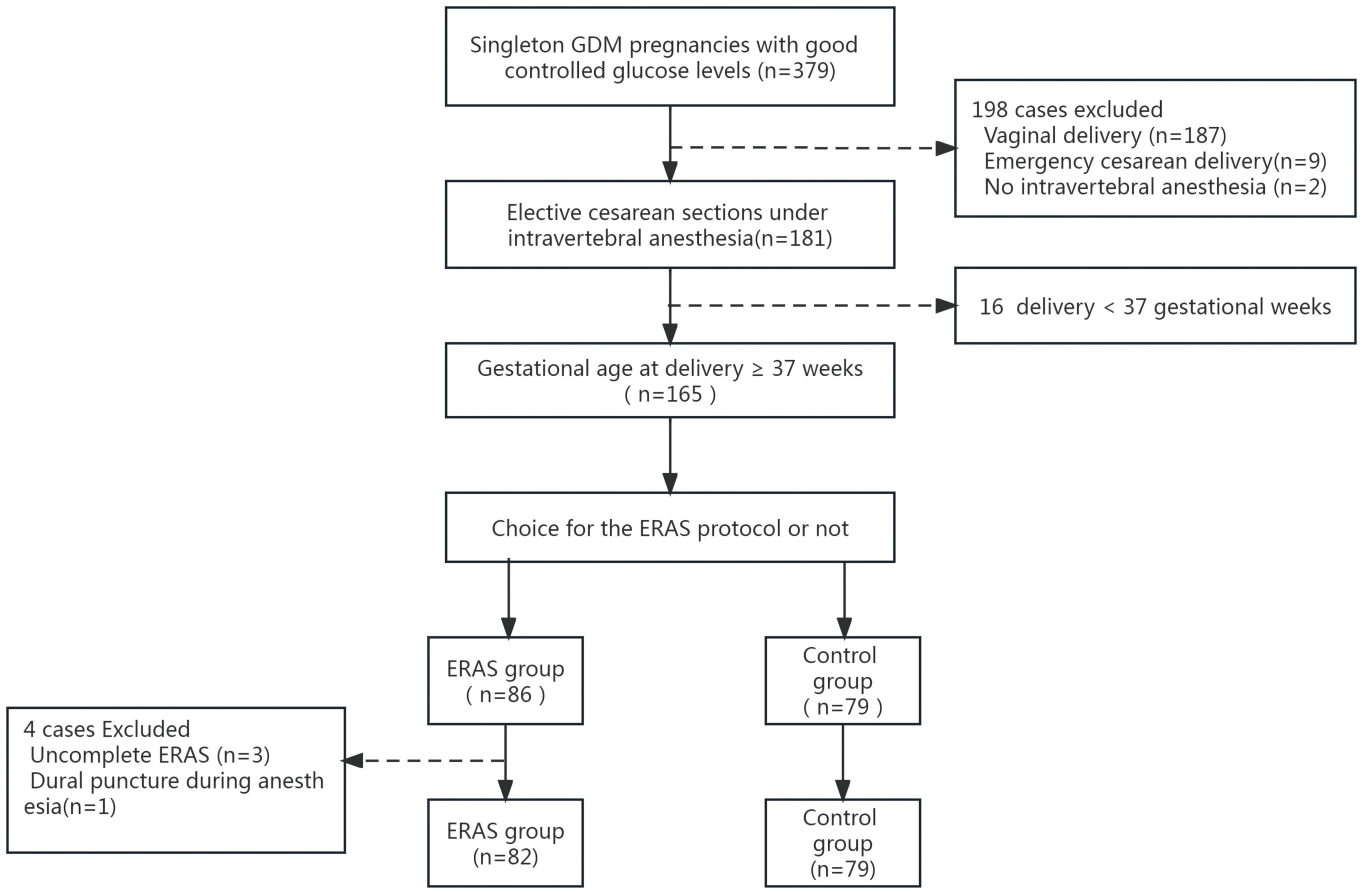
Flowchart of the study population. GDM, gestational diabetes mellitus.

A total of 165 female subjects were included in the study, including 86 in the ERAS group and 79 in the control group. However, exclusions occurred due to various reasons: in the ERAS group, 3 did not complete ERAS protocols, and 1 experienced a dural puncture during anesthesia. This resulted in 161 patients completing the analyses (82 in the ERAS group and 79 in the control group) (**Figure 1**). Demographic and clinical comparisons: The two groups showed no significant differences in demographic characteristics, medical comorbidities, or indications for cesarean section (**Table 1**).

**Table 1:** Maternal characteristics at baseline by group.

Baseline demographic	Total population (n = 161)	Control (n = 79)	ERAS (n = 82)	*P* value
Age, years	34.37 ± 4.26	33.73 ± 3.90	34.99 ± 4.53	0.06
Hypertensive disorder of pregnancy, n (%)	9 (5.59)	4 (5.06)	5 (6.1)	1
BMI, kg/m^2^	27.62 ± 2.74	27.46 ± 2.07	27.79 ± 3.27	0.45
Gestational age, weeks	38.64 ± 0.75	38.67 ± 0.72	38.61 ± 0.77	0.65
Primigravidae, n (%)	42 (26.09)	15 (18.99)	27 (32.93)	0.1
Primiparous, n (%)	47 (29.19)	20 (25.32)	27 (32.93)	0.29
Indications for CS, n (%)				0.2
Prior cesarean delivery	94 (58.39)	50 (63.29)	44 (53.66)	
Malpresentation	20 (12.42)	11 (13.92)	9 (10.98)	
Placenta previa	8 (4.97)	6 (7.59)	2 (2.44)	
Cephalopelvic disproportion	6 (3.73)	2 (2.53)	4 (4.88)	
Suspicious fetal distress	1 (0.62)	0 (0)	1 (1.22)	
Suspected macrosomia	10 (6.21)	4 (5.06)	6 (7.32)	
Primary elective	21 (13.04)	6 (7.59)	15 (18.29)	
GDM, n (%)				0.98
A1	98 (60.87)	48 (60.76)	50 (60.98)	
A2	63 (39.13)	31 (39.24)	32 (39.02)	
CS Time, hours	49.75± 16.50	50.91± 18.10	48.63± 14.84	0.38
Aspiration, n (%)	0	0	0	–
Intraoperative blood loss, ml	422.36 ± 171.11	422.78 ± 192.12	421.95 ± 149.30	0.98
Blood loss at 24 hours postpartum, ml	596.32 ± 175.43	586.59 ± 185.58	605.70 ± 165.67	0.49
PPH, n (%)	4 (2.48)	1 (1.27)	3 (3.66)	0.62
Birth weight, kg	3.23 ± 0.43	3.22 ± 0.40	3.25 ± 0.46	0.76
Low Apgar score, n (%)	0	0	0	–

GDM, gestational diabetes mellitus; CS, caesarean section; PPH, postpartum hemorrhage.

Continuous variables were shown as mean ± SD; Categorical variables presented as number (percentage).

*P* < 0.05 compared to control.

### 3.2 Perioperative blood glucose between the ERAS group and the control group

Preoperative glucose levels were significantly higher in the ERAS group (5.01 ± 1.06 mmol/L) compared to the control group (4.45 ± 0.90 mmol/L, *p*<0.001). In terms of clinical significance, the mean difference between the ERAS group and control group was 0.47 mmol/L (95% CI: 0.15-0.80 mmol/L) adjusted by maternal age, HPD, BMI, gestational age, primigravidae, primiparous, GDM, CS time, and fasting glucose, which is lower than the clinically meaningful difference of 1 mmol/L (**Table 2**).

**Table 2:** Linear regression analysis of blood glucose measures and ERAS.

Blood glucose (mmol/L)	Total (n = 161)	Control (n = 79)	ERAS (n = 82)	Treatment difference			Adjusted* model		
				β	95%CI	*P* values	β	95%CI	*P* values
Maternal									
Fasting	4.38 ± 0.64	4.27 ± 0.60	4.48 ± 0.66	0.2	0.01~0.4	0.041	0.18	-0.03~0.39	0.088
Preoperative	4.73 ± 1.02	4.45 ± 0.90	5.01 ± 1.06	0.56	0.26~0.87	<0.001	0.47	0.15~0.80	0.005
Postoperative	4.47 ± 0.73	4.31 ± 0.89	4.62 ± 0.49	0.31	0.09~0.53	0.007	0.25	0.02~0.48	0.037
Fasting on the postoperative day	3.99 ± 0.75	3.78 ± 0.60	4.18 ± 0.82	0.4	0.17~0.62	0.001	0.29	0.10~0.49	0.004
The differences in the levels of glucose from fasting to the perioperative									
Preoperative	0.35 ± 1.05	0.17 ± 0.91	0.53 ± 1.14	0.36	0.04~0.67	0.031	0.47	0.15~0.8	0.005
Postoperative	0.09 ± 0.83	0.04 ± 0.98	0.14 ± 0.66	0.11	-0.15~0.36	0.425	0.25	0.02~0.48	0.037
Fasting on the postoperative day	-0.39 ± 0.79	-0.49 ± 0.57	-0.30 ± 0.95	0.19	-0.05~0.44	0.126	0.29	0.10~0.49	0.004
Neonatal									
Birth	2.77 ± 0.49	2.55 ± 0.40	2.98 ± 0.47	0.43	0.3~0.57	<0.001	0.45	0.30~0.59	<0.001

Continuous variables were shown as mean ± SD.

*Adjusted by maternal age, hypertensive disorder of pregnancy (HPD), body mass index (BMI), gestational age, primigravidae, primiparous, gestational diabetes mellitus (GDM), caesarean section (CS) time and fasting glucose.

A comparative analysis of preoperative fasting blood glucose, immediate postoperative blood glucose, and fasting blood glucose on the first post-cesarean day revealed a significant increase in preoperative fasting glucose in the ERAS group compared to the control group. However, both immediate postoperative glucose and fasting glucose on the first post-cesarean day were significantly higher in the ERAS group. Notably, the glucose difference between the groups was less than 1 mmol/L (**Table 2**).

Due to concerns that preoperative carbohydrate loading in ERAS may exacerbate hyperglycemia, many healthcare providers currently do not recommend ERAS for diabetic patients. In this study, in contrast to the control group, the fasting glucose on the day of operation in the ERAS group increased by 0.2 mmol/L (95% CI 0.01-0.4 mmol/L). In the control group, the increase was 0.17 ± 0.91 mmol/l from fasting to preoperative glucose by traditional preoperative management (**Table 2**). According to a previous study, an increase of 1 mmol/L in maternal blood glucose might raise the risk of neonatal hypoglycemia. Therefore, in this study, an elevation in preoperative glucose of 1.5 mmol/L (1 mmol/L+0.2+ 0.2 ± 0.9 mmol/L≈1.5 mmol/L) in the ERAS group compared to the control group might raise the risk of neonatal hypoglycemia. Furthermore, our study contrasts the cases of elevation in glucose of 1.5 mmol/L from fasting to preoperational between the ERAS and the control groups. All the cases of elevation in glucose of 1.5 mmol/L from fasting to preoperational were 12, including 9 in the ERAS group and 3 in the control group. No statistically significant differences between the groups were found (P = 0.083) (**Supplementary Table 2**).

We also noticed that the highest value of preoperative glucose in the ERAS group was 10.4 mmol/l, but in the control group it peaked at 9.6 mmol/l, and the highest value difference between the ERAS group and control group was 0.8 mmol/L, which is lower than the clinically meaningful difference of 1 mmol/L.

Another important finding from this study was that neonatal glucose was significantly different in ERAS women (2.98 ± 0.47 mmol/L) compared with control women (2.55 ± 0.40 mmol/L). The ERAS group newborn led to an over 0.45 mmol/L (95% CI 0.31~0.59) elevation of the blood glucose level compared with the control group (*p* < 0.001) after adjustment by maternal age, HPD, BMI, gestational age, primigravidae, primiparous, GDM, CS time, and fasting glucose.

### 3.3 Safety evaluation of ERAS in contrast to the control group

**Figure 2:**
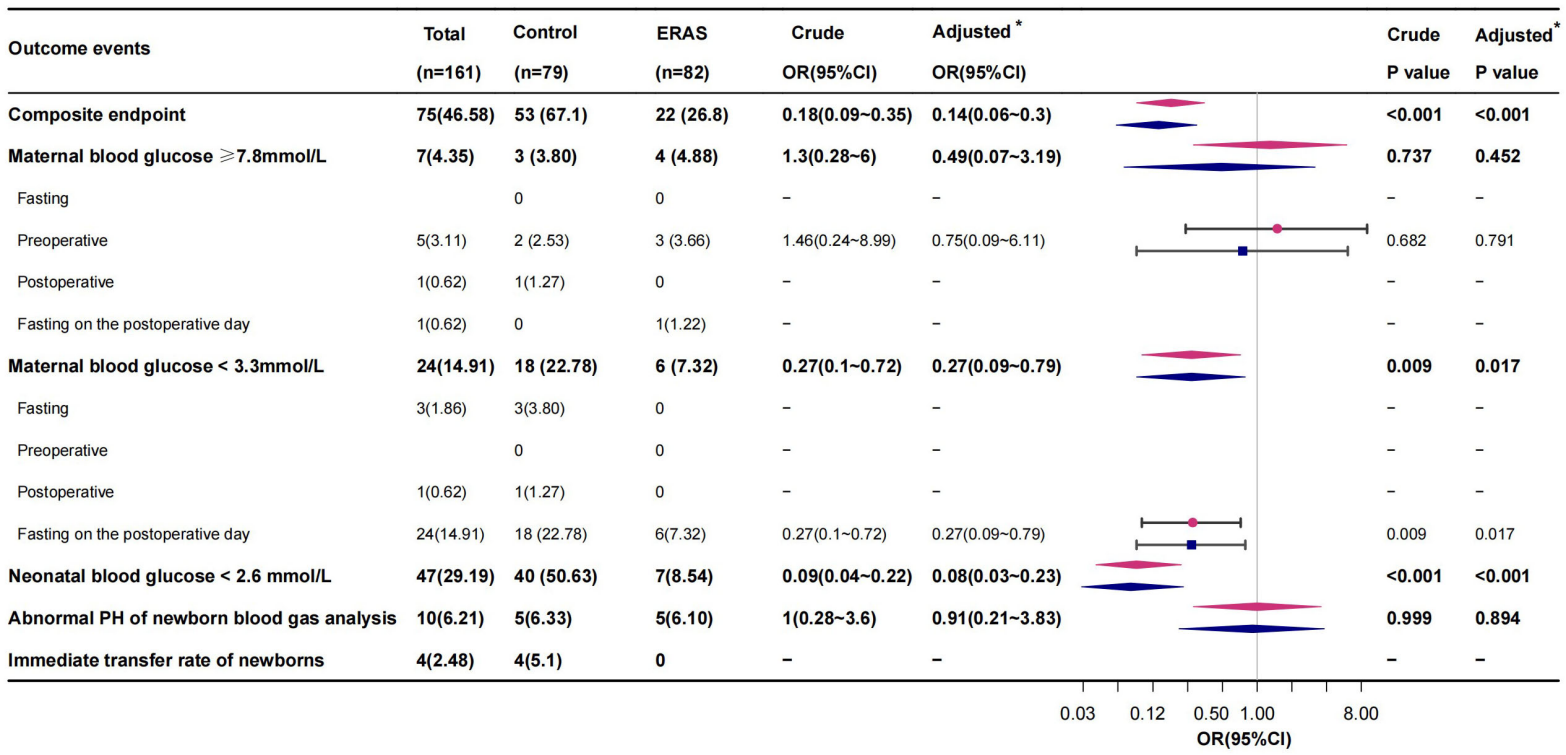
Logistics regression analysis of composite endpoints and endpoint composition in the study of GDM patients treated with ERAS. *Adjusted by maternal age, hypertensive disorder of pregnancy (HPD), body mass index (BMI), gestational age, primigravidae, primiparous, gestational diabetes mellitus (GDM), caesarean section (CS) time, and fasting glucose. Red represents the crude model, and blue represents the adjusted model.

In order to examine the safety of the ERAS program for patients with GDM, we built a composite adverse outcome including dysglycemia and dysglycemia-related adverse events in the perioperative setting and compared the groups (**Figure 2**, **Table 2**). A total of 22 cases (26.8%) of composite adverse outcomes occurred in the ERAS group, compared with 53 cases (67.1%) in the control group. The difference in composite adverse outcome between the two groups was mainly in the risk of perioperative maternal and neonatal hypoglycemia. Out of them, according to the diagnosis of hypoglycemia criteria, 6 (7.32%) gestational women and 7 (8.54) newborns were in the ERAS group, while 18 (22.78%) gestational women and 40 (50.63%) newborns were in the control group. After adjustments for maternal age, HPD, BMI, gestational age, primigravidae, primiparous, GDM, CS time, and fasting glucose, the ERAS group demonstrated an 86% (OR 0.14, 95% CI 0.06~0.3) reduction in the risk of composite adverse outcomes, including the risk of perioperative maternal hypoglycemia decreasing 73% (OR 0.27, 95% CI 0.09~0.79) and the rate of neonatal hypoglycemia decreasing 92% (OR 0.08, 95% CI 0.03~0.23) (**Figure 2** and **Table 2**). However, of equal importance is that the ERAS protocol did not significantly increase the risk of maternal hyperglycemia.

No cases of aspiration occurred in either group during the cesarean section. Additionally, there were no significant differences in other adverse outcomes, including abnormal umbilical artery blood gas values, low Apgar scores, or immediate postnatal pediatric transfer rates, between the groups’ neonates.

## 4 Discussion

This retrospective cohort study shows that ERAS protocols for cesarean deliveries in GDM patients who have good control of their blood sugar levels do not increase the risk of severe hyperglycemia before surgery. Additionally, it reduces the incidence of perioperative maternal and neonatal hypoglycemia, thereby providing substantial evidence for the application of ERAS in GDM scenarios.

A key benefit of ERAS is the preoperative administration of a preoperative clear drink (PCD) with a low carbohydrate content. This practice interrupts the overnight fasting metabolic state and induces an anabolic state through stimulation of endogenous insulin release (20). This approach not only stabilizes perioperative blood glucose levels but also diminishes postoperative complications (20). However, the application of ERAS in GDM patients before elective cesarean sections has been constrained by insufficient clinical research data, particularly concerning the impact of preoperative low-dose carbohydrate administration on blood glucose levels and the potential for severe glycemic irregularities.

Our study revealed significantly higher preoperative blood glucose levels in ERAS patients compared to control patients, which might be the primary concern for the current recommendation against using ERAS in GDM patients. However, in terms of clinical significance, the mean difference between the ERAS group and control group was significantly lower (*p*< 0.001) than the clinically meaningful difference of 1 mmol/L. ERAS protocol and traditional perioperative management were not significantly associated with the incidence of clinically meaningful amplitude of glycemic excursions (≥1 mmol/L). Therefore, the ERAS protocol would not lead to a clinically meaningful increase in preoperative blood glucose in pregnant women.

Our study findings were consistent with the results of previous similar studies. A prospective, non-inferiority cohort study evaluating preoperative carbohydrate loading in non-pregnant diabetic patients found no significant elevation in a surrogate marker for postoperative complications, namely preoperative blood glucose, and the observed difference in preoperative blood glucose values was also minimal (0.23 mmol/L, 95% CI: -1.00-1.45 mmol/L) (6). Furthermore, a randomized controlled prospective study demonstrated that in GDM patients with well-managed glycemia, an ERAS protocol with low-dose carbohydrates prior to elective cesarean sections maintained glycemic values comparable to the control group (21). Research by U. O. Gustafsson et al. on a non-pregnant cohort with uncomplicated type II diabetes also established the safety of preoperative carbohydrate-rich beverages, showing no increased risk of hyperglycemia or aspiration (22). Studies have shown that CHO deficiency results in a lack of building blocks for tissue regeneration and other anabolic processes. In addition, low CHO intake decreases CHO oxidation and may increase insulin resistance (23). Oral and enteral nutritional support is safe for patients with type 2 diabetes (23). Therefore, patients with GDM who maintain good glycemic control should consider the ERAS protocol.

In order to examine the safety of the ERAS program for patients with GDM, we further analyzed a composite adverse outcome including dysglycemia and dysglycemia-related adverse events in the perioperative. The ERAS protocol demonstrated an 86% reduction in composite adverse outcomes, including a 73% decrease in the risk of perioperative maternal hypoglycemia and a 92% decrease in the rate of neonatal hypoglycemia. Maternal and neonatal hypoglycemia were serious perioperative complications for pregnant women with GDM. In particular, neonatal hypoglycemia was particularly perilous as it impacts the metabolism and development of neonatal brain cells (24). Persistent hypoglycemia or significant blood glucose fluctuations can cause irreversible brain damage in neonates. Prior research indicated a high incidence (51%) of hypoglycemic events in newborns with associated risk factors (24). Pregnancy combined with diabetes mellitus is a predominant maternal risk factor for neonatal hypoglycemia (25). Neonates born by caesarean section are more likely to have low blood glucose levels, higher rates of neonatal infections, and more frequent admissions to the neonatal intensive care unit (NICU) than those born by vaginal delivery (26, 27). Our study proved that the ERAS protocol could significantly improve the incidence of hypoglycemia in newborns with GDM. The reason may be that the ERAS protocol can stabilize maternal preoperative blood glucose levels. Neonatal hypoglycemia is linked to both elevated and reduced preoperative maternal blood glucose levels for pregnant women with GDM (1). A certain level of maternal glucose before and after delivery is a protective factor against neonatal hypoglycemia at birth (28). Therefore, maintaining perioperative blood glucose levels within a stable and acceptable range is vital for reducing surgical complications. For pregnant women with GDM, stress hyperglycemia induced by traditional perioperative management should not be ignored. This traditional approach is thought to trigger a stress response due to prolonged fasting, which may disrupt insulin pathways by altering neuroendocrine and inflammatory responses, leading to perioperative insulin resistance, stress hyperglycemia, or perioperative diabetes (29).

Conversely, the preoperative administration of oral carbohydrates significantly mitigates insulin resistance and aids in stabilizing perioperative glycemic levels (30). The procedure triggers the body’s natural production of insulin and ends the metabolic state of fasting during sleep, leading to improved recovery after surgery (31). Previous findings suggest that ERAS implementation mitigates the risk of maternal starvation ketosis and hypoglycemia due to prolonged fasting, as documented in reference (2). Combined with the results of our study, we suggest the ERAS protocol might be more safe compared to traditional perioperative management for good glucose control in GDM patients.

In this study, the ERAS protocol significantly lowered the incidence of maternal hypoglycemia without increasing severe maternal hyperglycemia. The prevalence of other adverse outcomes, including abnormal umbilical arterial blood gas values, low Apgar scores, and the necessity for immediate postnatal transfer to a pediatric unit, did not differ significantly between the groups. The results coincided with previous studies. Firstly, for type 2 diabetes, preoperative carbohydrate-rich beverages did not increase the risk of hyperglycemia or misaspiration (22). Secondly, a low dose of carbohydrate-rich beverages cannot cause a clinically significant glucose difference (1 mmol/L), which is associated with neonatal hypoglycemia and other hypoglycemia-related perioperative complications. Oral administration of a carbohydrate-rich solution two hours before labor has been shown to independently increase blood glucose levels in infants (23). Furthermore, ERAS ensures adequate energy substrate availability for the fetus and diminishes the likelihood of neonatal hypoglycemia (32).Consequently, this approach contributes to improved neonatal prognosis and a reduction in adverse outcomes associated with hypoglycemia.

This study has several limitations. First, it relied on single-center data, necessitating validation through an expanded study population. Second, emergency cesarean-section cases were excluded due to uncontrolled fasting durations. Additionally, we excluded pre-pregnancy diabetics to mitigate the risk of excessive glycemic response to oral carbohydrates, focusing solely on GDM patients. Consequently, applying these findings to the broader diabetic pregnant population should be approached with caution. Third, determining the optimal carbohydrate dosage for pregnant women undergoing cesarean delivery remains unresolved. Total carbohydrate intake below 50 g was deemed a low dose (14), as per a referenced meta-analysis. We used a clarified beverage containing 42.6 g of carbohydrate with maltodextrin to enhance intestinal transport mechanisms, thereby expediting energy absorption and hydration while reducing gastric emptying time (15). This preparation also alleviates patient discomfort, including thirst, hunger, and anxiety (16). Future studies could use a multi-center, prospective study approach to include all pregnant women with gestational diabetes who undergo a caesarean section to determine the appropriate carbohydrate dose.

## 5 Conclusion

In patients with GDM and well-controlled blood glucose, an ERAS regimen incorporating a low-dose carbohydrate intake (42.6 g) two hours before elective cesarean sections did not lead to a clinically significant maternal glucose increase. Additionally, it significantly reduced the incidence of maternal and neonatal hypoglycemia and hypoglycemia-related perioperative complications. Consequently, we recommend a low-dose carbohydrate ERAS regimen for GDM patients with good glycemic control to improve hypoglycemia-related perioperative complications.

## Data availability statement

The datasets presented in this study can be found in online repositories. The names of the repository/repositories and accession number(s) can be found below: https://rdd.sysu.edu.cn/UserHome/ProjectNew.aspx.

## Ethics statement

The studies involving humans were approved by the Institute Medical Ethics Committee of the Third Affiliated Hospital of Sun Yat-sen University. The studies were conducted in accordance with the local legislation and institutional requirements. The participants provided their written informed consent to participate in this study. Written informed consent was obtained from the individual(s) for the publication of any potentially identifiable images or data included in this article.

## Author contributions

JZ: Conceptualization, Methodology, Writing – original draft. PZ: Methodology, Writing – original draft. ZT: Writing – review & editing. CL: Writing – review & editing. LY: Writing – review & editing. TH: Writing – review & editing. HH: Writing – review & editing. YY: Conceptualization, Methodology, Project administration, Writing – review & editing.
