# Supplementary material for: Enhanced recovery after surgery in elective cesarean section patients with gestational diabetes mellitus does not lead to glucose-related maternal and neonatal complications
Source: Front Endocrinol (Lausanne). 2024 Aug 6;15:1403754. doi: 10.3389/fendo.2024.1403754 (PMC11333357; doi:10.3389/fendo.2024.1403754)
Supplement: Supplementary file 1 [file Table_1.docx]

Supplemental Table 1 Management of ERAS protocol

| **Item** | **Key operation** |
| --- | --- |
| Assessment | Screen for GDM and assess optimization for surgery |
|  | Assess the risk of maternal and neonatal blood glucose abnormalities and adverse perinatal outcomes during the perioperative period |
| Education | ERAS pathway |
|  | Provide ERAS brochure and nutrition patient information |
| Diet | Take 52g of glucema before midnight the night before surgery |
|  | clear 14.2% carbohydrate drink until 2 hours prior to surgery |
| Day of surgery | |
| Preoperative | Multimodal pain management |
|  | Antibiotic prophylaxis cefuroxime 2 g IV within30- 60 minutes of incision |
| Intraoperative | Combined spinal-epidural anesthesia  During the operation, a warm infusion is carried out according to the actual situation, and a warm mattress is equipped to maintain a body temperature of 36-37 °C. Ensure that the temperature of the operating room is controlled at 24-26 degrees Celsius and the humidity is controlled at about 50%.  Peripheral glucose monitoring is performed every 30 minutes during anesthesia to guide intraoperative management based on the results  Goal-directed intraoperative fluid management  Delayed cord clamping for at least 1 minute at a term delivery  Skin-to-skin contact and breastfeeding are encouraged immediately after delivery |
| Postoperative | Multimodal analgesia  Use of multi-target combination antiemetics to reduce PONV occurrence  Resume oral eating and drinking as soon as possible, start a small amount of multiple eating liquids 2h after surgery, eat semi-liquid 6 ~ 24h after surgery, and general food after exhaust  Promote breastfeeding, early contact, early sucking, and early initiation |

Supplemental Table 2 The incidence rate of a 1.5 mmol/L increase in maternal perioperative glucose compared with fasting glucose

| perioperative glucose | Total(n = 161) | Control(n = 79) | ERAS(n = 82) | P values |
| --- | --- | --- | --- | --- |
| Preoperative | 12 ( 7.5) | 3 (3.8) | 9 (11) | 0.083 |
| [Postoperative](javascript:;) | 1 ( 0.6) | 0 (0) | 1 (1.2) | 1 |
| Fasting on the postoperative day | 1 ( 0.6) | 0 (0) | 1 (1.2) | 1 |

Categorical variables are presented as number (percentage)

P < 0.05 compared to control.
